# Short‐Term Results of the SONCAR Study: Optimized Neoadjuvant Chemoradiotherapy in Locally Advanced Rectal Cancer Patients

**DOI:** 10.1002/mco2.70222

**Published:** 2025-07-23

**Authors:** Rongxin Zhang, Fulong Wang, Xinhua Jiang, Hao Wang, Weili Zhang, Zhifan Zeng, Yuanhong Gao, Xiaojun Wu, Gong Chen, Liren Li, Peirong Ding, Shixun Lu, Jian Zhang, Min Liu, Qiaoxuan Wang, Weiwei Xiao, Zhizhong Pan, Desen Wan, Zhen‐hai Lu

**Affiliations:** ^1^ Department of Colorectal Surgery Sun Yat‐sen University Cancer Center Guangzhou Guangdong China; ^2^ State Key Laboratory of Oncology in South China Guangzhou Guangdong China; ^3^ Guangdong Provincial Clinical Research Center for Cancer Guangzhou Guangdong China; ^4^ Department of Radiology Sun Yat‐sen University Cancer Center Guangzhou Guangdong China; ^5^ Department of Radiation Sun Yat‐sen University Cancer Center Guangzhou Guangdong China; ^6^ Department of Pathology Sun Yat‐sen University Cancer Center Guangzhou Guangdong China; ^7^ State Key Laboratory of Ophthalmology Zhongshan Ophthalmic Center Sun Yat‐sen University Guangzhou Guangdong China; ^8^ Department of Ultrasound Sun Yat‐sen University Cancer Center Guangzhou Guangdong China

**Keywords:** locally advanced rectal cancer, neoadjuvant chemoradiotherapy, oxaliplatin, toxicity

## Abstract

This research endeavored to ascertain whether four cycles of oxaliplatin in conjunction with standard radiation (oxaliplatin‐CRT) could enhance overall survival when compared with standard neoadjuvant chemoradiotherapy (nCRT) in locally advanced rectal cancer (LARC). A Phase III randomized trial (SONCAR Trial, NCT02031939) was conducted in China, involving patients diagnosed with clinical T3‐4 and/or N+ rectal cancer. Patients were randomly allocated to the experimental arm (receiving pelvic radiation (50 Gy/25 fractions) in conjunction with oxaliplatin and capecitabine) or the control arm (pelvic radiation in conjunction with capecitabine alone). The main endpoint was a 5‐year OS, while the secondary objectives encompassed pathological complete response (pCR), 3‐year disease‐free survival, and surgical complications. A total of 536 patients were assessable. The rate of pCR was notably higher in the experimental group (31.9%) than in the control group (21.5%) (*p* = 0.008). The clinical complete response (cCR) rate was also higher in the experimental group (*p* = 0.024). Among patients with tumors located within 5 cm of the anal verge, the experimental group exhibited a significantly greater tumor regression, with rates of 33.8% compared to 21.6% in the control group (*p* = 0.024). In summary, oxaliplatin‐CRT significantly augmented the tumor response in LARC patients with manageable toxicity.

## Graphical abstract

This study investigates whether adding four cycles of CapOX to standard neoadjuvant chemoradiotherapy improves overall survival in patients with locally advanced rectal cancer. The results show that this combination significantly increases complete tumor response rates, particularly for tumors close to the anal verge, with manageable toxicity.

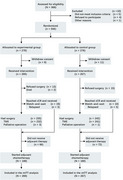

## 1 Introduction

Rectal cancer constitutes one of the most prevalent cancers in China [1]. In the past, patients with locally advanced rectal cancer (LARC) faced a significant risk of local recurrence, poor sphincter preservation, a low long‐term survival rate, and a considerable rate of distant metastasis. Previous studies, including CAO/ARO/AIO‐04 [2], ACCORD‐12 [3], and NSABPR‐04 [4], reported that neoadjuvant chemoradiotherapy (nCRT) reduces the local failure rate to less than 5%. In contemporary clinical practice, the conventional approach for LARC involves nCRT, which is succeeded by total mesorectal excision (TME). However, long‐term survival and sphincter preservation have not been improved. The evaluation of clinical complete response (cCR) after nCRT is highly significant for patients with very low rectal cancer. It can offer more patients the chance to adopt the watch‐and‐wait strategy [5], which is one of the most important ways for sphincter preservation. The application of diffusion‐weighted magnetic resonance imaging (DWI) helped more LACR patients to undergo noninvasive and accurate evaluation of tumor response, which facilitated the accurate identification of cCR and enabled organ preservation through the watch‐and‐wait strategy. Nevertheless, it is noted that the commonly used standard nCRT (capecitabine combined with pelvic radiation) can only achieve a pathological complete response (pCR) rate of less than 20%, and distant metastasis still occurs in 30% of patients [6]. There are still many challenges in improving the outcomes of nCRT.

Previous investigations have divulged that the pCR rate subsequent to standard chemoradiotherapy (CRT) approximates 15% [7]. The incorporation of a second drug might engender a superior response and enhance long‐term survival. Oxaliplatin (OXA) has been integrated into CRT in numerous clinical trials; however, the results remain contentious. In the STAR‐01 [8], NSABP R‐04 [4], and ACCORD 12/0405 [9] trials, the incorporation of OXA did not augment the pCR rate or long‐term survival and only enhanced toxicity. On the contrary, the findings of the German CAO/ARO/AIO‐04 [10] and Chinese FOWARC [7] trials indicated that the incorporation of OXA to fluorouracil RT elevated the pCR rate (17% vs. 13%, *p* = 0.038) and the disease‐free survival (DFS) rate in the AIO‐04 trial (75.9% vs. 71.2%). Nevertheless, whether OXA ought to be incorporated into nCRT remains a subject of debate. A phase III clinical trial demonstrated that in UGT1A1 genotype 11 or 12 LARC, irinotecan combined with capecitabine‐based CRT raised the pCR rate [11]. When comparing the control cohort, the pCR rate was 15% in the control cohort and increased to 30% in the experimental cohort (risk ratio: 1.96; 95% CI, 1.30–2.97; *p* < 0.001). Nonetheless, the evidence concerning the utilization of irinotecan in nCRT remains limited.

The second approach to enhancing the pCR rate and survival is the total neoadjuvant chemoradiotherapy (TNT) strategy. In accordance with the outcomes of RAPIDO, the TNT method is an effective means to improve the pCR rate for LARC (27.7% vs. 13.8% in the short‐course arm and long‐course arm, respectively; odds ratio [OR] 2.40; *p* < 0.001) [12]. A greater number of patients who achieved cCR could achieve organ and bowel function preservation through the watch‐and‐wait strategy. Furthermore, nCRT employing the TNT approach can improve long‐term survival; however, more severe adverse events (AEs) were reported (38%) [12]. Consequently, the number of chemotherapy cycles that should be administered to enhance the pCR rate and long‐term survival with the least severe AEs remains undetermined.

The considerable progress of immunotherapy in the treatment of solid tumors has led to numerous studies suggesting that the utilization of PD‐1 inhibitors as a single therapy in patients with mismatch repair‐deficient (dMMR) rectal cancer can achieve a complete response (CR) rate as high as 75%, thereby enabling organ preservation [13]. In 2023, the NCCN (National Comprehensive Cancer Network) and the CSCO (Chinese Society of Clinical Oncology) proposed this approach as a consensus [14, 15]. Although PD‐1 monotherapy has been demonstrated to be nearly ineffective in patients with mismatch repair‐proficient (pMMR) rectal cancer, prior studies have disclosed a synergistic impact when combining CRT with PD‐1/PD‐L1 inhibitors [16, 17]. A prospective study presented by Xu et al. suggested that the cCR and pCR were conspicuously in the cohort receiving nCRT in combination with PD‐1 inhibitors in contrast to the group receiving only nCRT (44.8% vs. 26.7%, *p* = 0.013) [18].

The study was designed to ascertain whether four cycles of OXA in combination with standard capecitabine‐based CRT could enhance the outcome of nCRT in LARC patients. The primary endpoint of this trial was to improve the 5‐year overall survival (OS) compared to standard nCRT. We also anticipated that this novel treatment strategy could increase the tumor response rate and organ preservation rate, lower the risk of distant metastases, and enhance survival while ensuring locoregional control. In this present study, we reported the safety of the experimental treatment, the pCR rate, treatment‐related toxicities, and postoperative complications in relation to the standard treatment.

## 2 Results

### 2.1 Study Participants and Neoadjuvant Therapy

**FIGURE 1:**
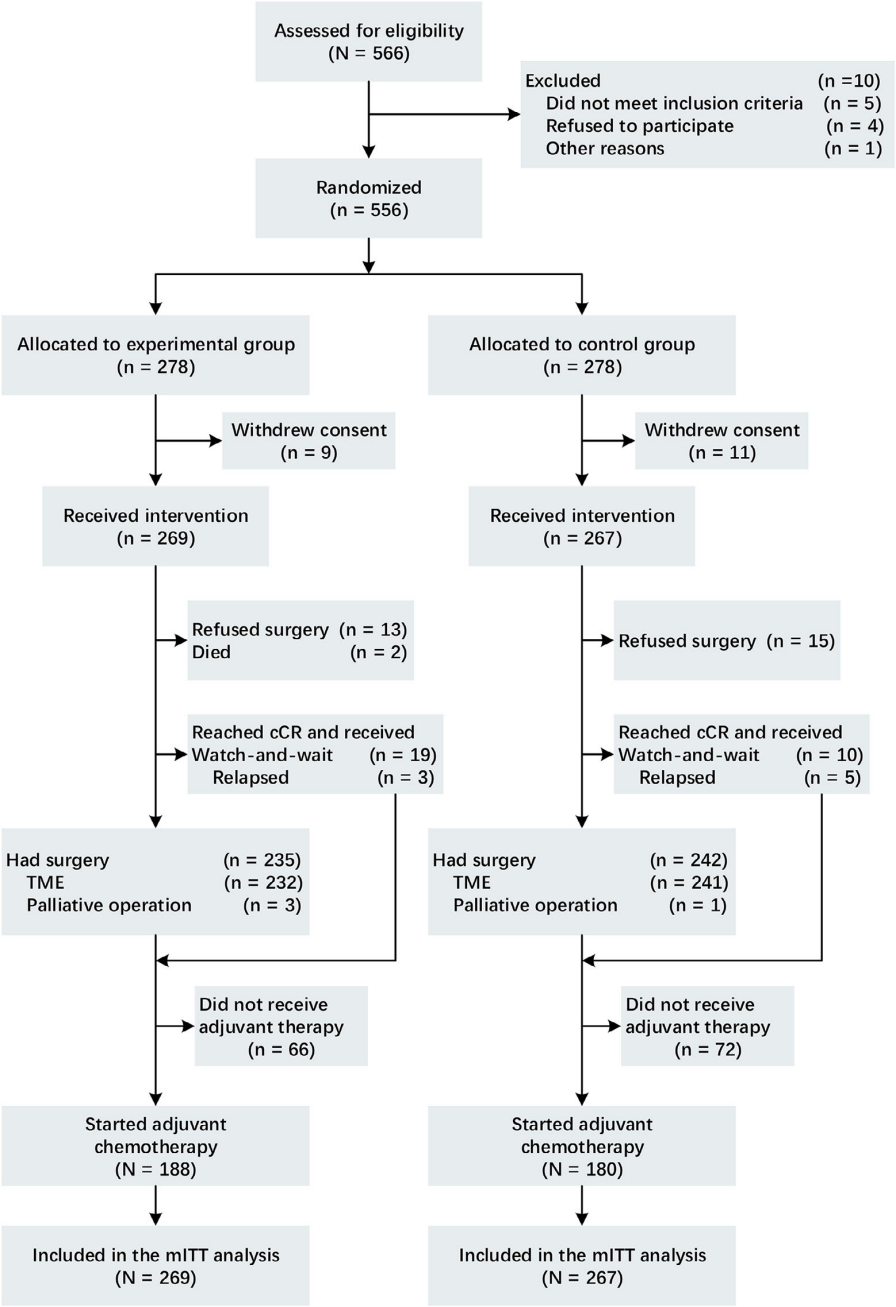
CONSORT diagram. The ITT population comprised all patients who were randomized to treatment (278 in both groups). The mITT population comprised all patients who were randomized to treatment and received intervention. TME, total mesorectal excision; cCR, clinical complete response; ITT, intention‐to‐treat; mITT, modified intention‐to‐treat.

From January 2014 to June 2020, 556 patients were enlisted in this study, with 278 patients assigned to each group (Figure 1). Specifically, 269 patients in the experimental group and 267 patients in the control group were assessable. Following randomization, 20 patients (9 in the experimental group and 11 in the control group) were excluded as they withdrew their consent to participate prior to receiving any treatment. Consequently, the remaining 536 patients formed the modified intention‐to‐treat (mITT) population, substituting the ITT population for further analysis. All baseline patient characteristics were evenly balanced (Table 1). MRI was employed to ascertain the T and N stages prior to nCRT. A total of 109 in the experimental group and 84 patients in the control group presented with mesorectal fascia (MRF)+ (constituting 40.7% and 31.5%, respectively), whereas 102 patients in the experimental group and 90 patients in the control group exhibited extramural vascular invasion (EMVI+) (accounting for 37.9% and 33.7%, respectively).

**TABLE 1:** Baseline characteristics of patients.

Characteristic	Treatment group, no. (%)	
	Experimental group (*n* = 269)	Control group (*n* = 267)
Age, years		
Mean (SD)	55.2 (10.6)	55.2 (10.2)
Median (range)	57 (26–75)	57 (27–75)
Sex		
Male	164 (61.0)	174 (65.2)
Female	105 (39.0)	93 (34.8)
ECOG performance Status		
0	242 (90.0)	240 (89.9)
1	27 (10.0)	27 (10.1)
BMI, kg/m^2^		
Mean (SD)	22.6 (3.1)	22.8 (2.9)
Median (range)	22.2 (15.7–33.5)	22.9 (15.3–33.3)
Clinical T category		
cT1	1 (0.4)	0
cT2	3 (1.1)	4 (1.5)
cT3	175 (65.8)	171 (64.0)
cT4	87 (32.7)	92 (34.4)
Clinical N category		
cN0	35 (13.0)	28 (10.5)
cN1	114 (42.4)	132 (49.4)
cN2	120 (44.6)	107 (40.1)
Distance from the anal verge, cm		
≤ 5 cm	170 (63.2)	156 (58.4)
> 5 cm	99 (36.8)	111 (41.6)
MRF		
Positive	109 (40.7)	84 (31.5)
Negative	141 (52.6)	167 (62.5)
Not reported	18 (6.7)	16 (6.0)
Levator ani involved		
Yes	1 (0.4)	2 (0.7)
No	268 (99.6)	265 (99.3)
External sphincter muscle of anus involved		
Yes	5 (1.9)	4 (1.5)
No	264 (98.1)	263 (98.5)
EMVI		
Positive	102 (37.9)	90 (33.7)
Negative	153 (56.9)	165 (61.8)
Not reported	14 (5.2)	12 (4.5)
Baseline CEA level		
Normal	156 (58.0)	139 (52.1)
Abnormal	103 (38.3)	116(43.4)
Unknown or missing	10 (3.7)	12 (4.5)
Baseline CA199 level		
Normal	197 (73.2)	215 (80.5)
Abnormal	44 (16.4)	39 (14.6)
Unknown or missing	28 (10.4)	13 (4.9)

Abbreviations: CA199, carbohydrate antigen 199.; CEA, carcinoembryonic antigen; ECOG, eastern cooperative oncology group; EMVI, extramural vascular invasion; MRF, mesorectal fascia; SD, standard deviation.

### 2.2 Neoadjuvant Treatment and Response Evaluation

A total of 257 (95.5%) patients in the experimental group and 252 (94.4%) in the control group received the complete dose of radiation. In the experimental group, 259 patients underwent one cycle of induction chemotherapy (258 patients received capecitabine plus oxaliplatin [CapOX], and 1 patient received capecitabine), while 10 patients did not receive induction chemotherapy. A total of 267 patients in the experimental group and 265 patients in the control group received chemotherapy during radiation. Moreover, 230 patients in the experimental group and 26 in the control group received consolidation chemotherapy after radiation therapy (more particulars are provided in Table S1). A total of 188 patients in the experimental group and 80 patients in the control group received adjuvant chemotherapy. Throughout the entire treatment period, 230 patients (85.5%) in the experimental group and 260 patients (97.4%) in the control group completed the full dose of neoadjuvant chemotherapy, while 172 patients (63.9%) in the experimental group and 110 patients (41.2%) in the control group completed the full cycles of adjuvant chemotherapy.

MRI was also utilized to determine the response to CRT. After nCRT, 67 patients (24.9%) in the experimental group and 64 (24.0%) in the control group remained MRF+. Additionally, 59 patients (21.9%) in the experimental group and 63 (23.6%) in the control group persisted as EMVI+. Compared with the situation before CRT, patients in both groups achieved disease regression (Table S2). Nevertheless, 7 patients in the experimental group and 12 patients in the control group developed distant metastases after nCRT (2.6% vs. 4.5%, *p* = 0.253).

### 2.3 Surgical Outcomes

In the experimental group, 235 patients (87.4%) underwent surgery, while in the control group, 242 patients (90.6%) underwent the surgical procedure (Table 2). The median duration between cardiac resynchronization therapy (CRT) and surgical intervention was observed to be 60 days for the experimental cohort, with a variation spanning from 40 to 222 days. In contrast, the control group exhibited a median interval of 59 days, which ranged from 35 to 604 days (Table S1). A total of 257 patients in the experimental group and 235 in the control group had R0 resections (95.7% vs. 97.1%, *p* = 0.693). Regarding laparoscopic surgery, 162 patients from the experimental group and 152 from the control group were included. Concerning anterior resection (AR), 176 in the experimental group and 182 in the control group underwent this operation. Both groups exhibited similar sphincter preservation rates (75.3% compared to 75.6%, *p* = 0.939). Temporary or permanent stoma formation was performed in 116 experimental group patients and 112 control group patients. No substantial differences in surgery duration or blood loss were noticed between the groups (3.6 h vs. 3.8 h and 50 mL vs. 50 mL, respectively). Postoperative complications occurred in 37 patients (15.7%) in the experimental group and 27 patients (11.2%) in the control group, yet the incidence did not significantly vary between the groups (*p* = 0.142). One patient in the experimental group passed away due to chemotherapy‐related adverse reactions. Anastomotic leakage occurred in 13 patients in the experimental group and 8 in the control group. All participants underwent a second ileostomy procedure. Two patients in the experimental group experienced anastomotic bleeding, while no patients in the control group had this complication. Further particulars are presented in Table 2.

**TABLE 2:** Surgical procedures and surgical complications.

	Treatment group, no. (%)		
Characteristic	Experimental group (*n* = 235)	Control group (*n* = 242)	*p* value
Surgery procedure			0.623
Anterior resection	176 (74.9)	182 (75.2)	
Abdominoperineal resection	50 (21.3)	56 (23.1)	
Hartmann	5 (2.1)	2 (0.8)	
Local resection	1 (0.4)	1 (0.4)	
Stoma only	3 (1.3)	1 (0.4)	
Temporary or permanent stoma			0.561
Yes	116 (49.4)	112 (46.3)	
No	119 (50.6)	130 (53.7)	
Sphincter preservation			0.939
Yes	177 (75.3)	183 (75.6)	
No	58 (24.7)	59 (24.4)	
Status of Surgery			0.693
R0 resection	225 (95.7)	235 (97.1)	
R1 resection	8 (3.4)	6 (2.5)	
R2 resection	2 (0.9)	1 (0.4)	
Surgery type			0.189
Laparoscopic	162 (68.9)	152 (62.8)	
Open	73 (31.1)	90 (37.2)	
Surgery time (hours)			0.628
Mean (SD)	3.9 (0.9)	3.9 (0.9)	
Median (Range)	3.6 (1.0–8.0)	3.8 (2.0–13.0)	
Blood loss during surgery (ml)			0.898
Mean (SD)	71.6 (5.5)	69.0 (3.4)	
Median (Range)	50 (10–800)	50 (5–400)	
Side effects of surgery			
Any complication	37 (15.7)	27 (11.2)	0.142
Anastomotic leakage	13 (5.5)	8 (3.3)	0.336
Anastomotic bleeding	2 (0.9)	0	0.466
Abdominal infection	1 (0.4)	0	0.988
Bowel obstruction	5 (2.1)	1 (0.4)	0.205
Infection of incisional wound	1 (0.4)	0	0.988
Dysuria	5 (2.1)	3 (1.2)	0.690
Bleeding	7 (3.0)	14 (5.8)	0.201
Pulmonary embolism	1 (0.4)	0	0.990
Renal function disorder	1 (0.4)	0	0.990
Lymphatic leakage	1 (0.4)	1 (0.4)	> 0.999
Clavien‒Dindo grade^a^			0.199
I	13 (5.5)	14 (5.8)	
II	7 (3.0)	4 (1.7)	
III	17 (7.2)	9 (3.7)	
IIIa	4 (1.7)	0	
IIIb	13 (5.5)	9 (3.7)	

Abbreviation: SD, standard deviation.

^a^ No patients were Clavien‒Dindo Grade IV or V.

### 2.4 Treatment Response and Oncological Outcomes

In the experimental group, pCR was witnessed in 75 patients, constituting 31.9%, while in the control group, it was observed in 52 patients, accounting for 21.5% (*p* = 0.008) (Table 3). In the experimental group, five patients were identified with progressive disease (PD), and in the control group, four patients were diagnosed with the same. Thirteen patients in the experimental group and 15 in the control group declined surgery for diverse reasons, encompassing personal factors and organ preservation (risks of APR) (Figure 1). A total of 19 patients in the experimental group and 10 patients in the control group attained cCR and opted for a watch‐and‐wait approach. Nevertheless, eight patients underwent local recurrence within 12 months after surgery and underwent salvage surgery resection (three in the experimental group and five in the control group, with respective rates of 15.8% and 50%). Consequently, the rate of cCR was 5.9% (16 of 269) for the experimental group and 1.9% (5 of 267) for the control group (*p* = 0.024). Furthermore, the tumor CR rate (pCR + cCR) was 33.8% (91 of 269) in the experimental group, which was significantly higher than that in the control group (21.3% [57 of 267]; *p* = 0.001).

**TABLE 3:** Pathologic characteristics of patients who underwent surgery.

	Treatment group, no. (%)		
Characteristic	Experimental group (*n* = 235)^a^	Control group (*n* = 242)^a^	*p* value
Tumor regression grade			0.007
0	75 (31.9)	52 (21.5)	
1	65 (27.7)	57 (23.6)	
2	88 (37.4)	123 (50.8)	
3	4 (1.7)	9 (3.7)	
Pathologic T category			0.006
ypT0	75 (31.9)	54 (22.3)	
ypT1	9 (3.8)	22 (9.1)	
ypT2	45 (19.1)	70 (28.9)	
ypT3	79 (33.6)	72 (29.8)	
ypT4	24 (10.2)	23 (9.5)	
Pathologic N category			0.474
ypN0	194 (82.6)	199 (82.2)	
ypN1	35 (14.9)	35 (14.5)	
ypN2	3 (1.3)	7 (2.9)	
ypStage			0.003
0	75 (31.9)	52 (21.5)	
I	45 (19.1)	78 (32.2)	
II	72 (30.6)	62 (25.6)	
III	35 (14.9)	38 (15.7)	
IV	5 (2.1)	11 (4.5)	
Pathologic complete response			0.008
Yes	75 (31.9)	52 (21.5)	
No	157 (66.8)	189 (78.1)	
Tumor deposit			0.927
Presence	11 (4.7)	11 (4.6)	
Absence	221 (95.3)	230 (95.4)	
Nerve vascular invasion			0.167
Presence	15 (6.5)	24 (10.0)	
Absence	217 (93.5)	217 (90)	
Vessel carcinoma embolus			0.412
Presence	13 (5.6)	18 (7.5)	
Absence	219 (94.4)	223 (92.5)	

^a^ Three patients in the experimental group and one patient in the control group received only stoma.

### 2.5 Adverse Events

In the experimental group, 58 (21.6%) patients presented with Grades 3–4 toxicities, while in the control group, 25 (9.4%) patients had such toxicities (*p* < 0.001) (Table 4). The prevalent Grades 3–4 toxicities encompassed anemia, thrombocytopenia, and leukopenia. In the induction chemotherapy stage, five patients in the experimental group underwent Grades 3–4 AEs. In the concurrent CRT period, 41 patients in the experimental group and 8 patients in the control group manifested Grades 3–4 AEs. During the consolidation chemotherapy phase, 18 patients in the experimental group and 1 patient in the control group encountered Grades 3–4 AEs.

**TABLE 4:** Chemotherapy‐related adverse event among patients.

	Treatment group, No. (%)		
Grade 3–4 Adverse Events	Experimental group (*n* = 269)	Control group (*n* = 267)	*p* value
Overall toxicity (NCI‐CTC version 4.0)	52 (19.3)	21 (7.9)	< 0.001
Hematologic			
Anemia	17 (6.3)	12 (4.5)	0.457
Thrombocytopenia	19 (7.1)	6 (2.2)	0.015
Leukopenia	11 (4.1)	4 (1.5)	0.120
Neutropenia	3 (1.1)	0	0.250
GI			
Vomiting	1 (0.4)	0	> 0.999
General AE			
Liver injury	1 (0.4)	0	> 0.999
Renal injury	1 (0.4)	0	> 0.999

### 2.6 Subgroup Analysis of Tumor Regression

**FIGURE 2:**
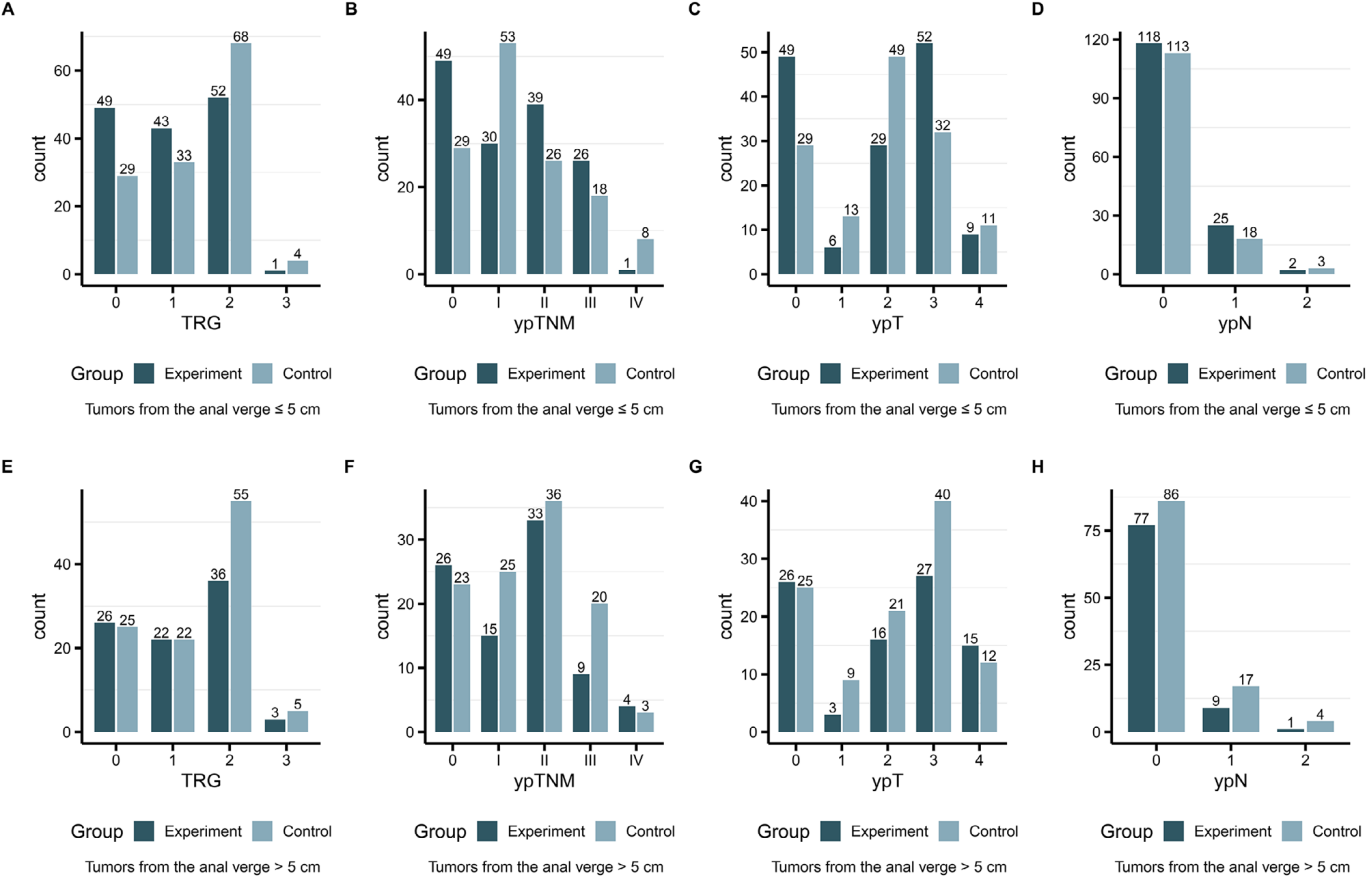
The relationship between distance from the tumor to the anal verge and tumor response. (A) TRG of tumors with distance ≤ 5 cm from the anal verge; (B) TRG of tumors with distance > 5 cm from the anal verge; (C) ypTNM stage of tumors with distance ≤ 5 cm from the anal verge; (D) ypTNM stage of tumors with distance > 5 cm from the anal verge; (E) ypT stage of tumors with distance ≤ 5 cm from the anal verge; (F) ypT stage of tumors with distance > 5 cm from the anal verge; (G) ypN stage of tumors with distance ≤ 5 cm from the anal verge; (H) ypN stage of tumors with distance > 5 cm from the anal verge. TRG, tumor regression grade.

In the ≤5 cm group, a significantly greater proportion of TRG0 (33.8% as opposed to 21.6%, *p* = 0.024; Figure 2A and Table S3), ypT0 (33.8% in contrast to 21.6%, *p* = 0.024; Figure 2E and Table S3), and pCR (33.8% compared to 21.6%, *p* = 0.024; Table S3) was noted. Nevertheless, the proportion of ypN0 did not show a significant difference between the two subgroups (81.4% vs. 84.4%, *p* = 0.514; Figure 2G and Table S3). However, in the > 5 cm group, no statistically significant dissimilarities in the proportions of ypT0, TRG0, pCR, or ypN0 were detected between the two subgroups (all *p* > 0.05; Figure 2B,D,F,H, and Table S3).

## 3 Discussion

Sphincter preservation constitutes a significant concern for patients with low rectal cancer. nCRT can enable more rectal cancer patients to attain sphincter preservation under a watch‐and‐wait approach. Enhancing the response rates of neoadjuvant chemotherapy will facilitate more patients in achieving cCR status. The addition of a second drug is the prime concern for all oncologists; nevertheless, in numerous trials, except for the AIO‐04 and FORWAC trials, OXA did not lead to any improvement in the pCR rate. These studies have faced criticism due to the low proportion of patients receiving the intended doses of chemotherapy as a consequence of postoperative morbidity. Most preceding studies employed OXA in a weekly regimen in nCRT, which did not notably enhance the pCR rate. The addition of OXA as a second drug during neoadjuvant RCT remains a subject of debate and is not recommended by the NCCN or ESMO guidelines [19]. To address compliance issues, we administered four cycles of chemotherapy, and OXA was incorporated into nCRT in a 3‐week regimen in the experimental group prior to surgery. Approximately 230 patients (85.5%) in the experimental group and 260 (97.4%) patients in the control group accomplished the full dose of neoadjuvant chemotherapy, and 172 (63.9%) and 110 patients (41.2%) completed full cycles of adjuvant chemotherapy. A greater number of patients were capable of completing full‐dose chemotherapy before surgery, indicating that four cycles of chemotherapy might enhance long‐term survival.

Another approach to boost the response rate is via the TNT strategy. In the OPRA trial, all rectal cancer patients underwent TNT treatment, and those who achieved pCR or near pCR adopted a watch‐and‐wait strategy. The 18‐month non‐TME survival rate was 78%. However, Grade 3+ AEs with systemic chemotherapy were 41% and 34% in the two groups, respectively [20]. The TNT model typically comprises six to eight cycles of chemotherapy; a higher number of chemotherapy cycles can result in more AEs. Hence, in this trial, we endeavored to increase the response rate while maintaining a low incidence of AEs. The result of this study indicated that more patients in the experimental group could achieve pCR status than those in the control group (31.9% vs. 21.5%; *p* = 0.008). Furthermore, the CR rate (pCR + cCR) in the experimental group was higher (33.8% as opposed to 21.3%, *p* = 0.001). The combination of CapOX and radiation therapy might imply a potential for increased pCR rates and improved watch‐and‐wait strategy results. It is notable that there was no substantial difference in the occurrence of surgical complications between the two groups (15.7% compared with 11.2%; *p* = 0.142), and the overall complication rate remained relatively low. Although the experimental group presented a higher incidence of Grade 3 or higher toxicity than the control group (21.6% vs. 9.4%, *p* < 0.001), the observed toxicity rate of 21.6% in the experimental group is lower than those reported in other comparable TNT regimens, such as the AIO‐12 trial (22%–37%), RAPIDO trial (34%–48%), and OPRA trial (34%–41%). Hence, we deem the associated toxicity in the experimental group of this study to be acceptable, particularly when considering the higher CR rate achieved.

In addition to noting satisfactory pathological outcomes, the experimental group also attained comparable results for the perioperative distant metastasis rate (metastases identified preoperatively and intraoperatively) (2.6% vs. 4.5%, *p* = 0.253), temporary or permanent stoma rate (49.4% as against 46.3%, *p* = 0.561), sphincter preservation rate (75.3% in contrast to 75.6%, *p* = 0.939), and R0 resection rate (95.7% compared to 97.1%, *p* = 0.693) to the control group. These short‐term outcomes further imply the safety and effectiveness of the treatment regimen employed in the experimental group. We also observed that after nCRT, the two groups had similar rates of EMVI and MRF positivity. Prior investigations have established that sustained positivity for EMVI following neoadjuvant therapy serves as a negative prognostic indicator for OS outcomes [21].

Significantly, in further subgroup analysis, the patients in the ≤ 5 cm group (that is, those whose tumors were less than or equal to 5 cm from the anal verge) responded more favorably to experimental CRT than those in the > 5 cm group. This might be more beneficial to patients with unresectable low rectal cancer, as they can achieve better tumor regression through nCRT, thereby reducing the difficulty of surgery and obtaining a better survival prognosis. This finding is also in line with the conclusions derived from the CONVERT and PROSPECT studies [22, 23]. The CONVERT study suggested a higher possibility of MRF involvement in very low rectal cancer, where the use of nCT alone has shown limited tumor regression. Consequently, the adoption of nCT in such patients is not advisable. Similarly, in the PROSPECT trial, patients with high‐risk factors such as T4 tumors were excluded and not recommended to undergo nCT. Our subgroup analysis further indicated that an intensified neoadjuvant treatment regimen might be more appropriate for very low rectal tumors, while patients with low to moderate rectal cancer and lower risk can avoid excessive use of nCRT.

The time interval from the conclusion of nCRT to surgical resection holds a crucial part in deciding the rates of pCR and cCR. Two investigations disclosed that a more extended waiting period led to a higher pCR rate in contrast to shorter intervals [24, 25]. A meta‐analysis integrating data from four trials and 22 additional nonrandomized studies (amounting to 25,445 patients) demonstrated that an 8‐week or longer gap between the completion of neoadjuvant RT and surgery was linked with higher probabilities of pCR (OR 1.41; 95% CI, 1.30–1.52) and tumor downstaging (primarily T stage, OR 1.33; 95% CI, 1.04–1.72) [26]. In our research, all patients in both groups waited for 6–8 weeks after nCRT. No substantial dissimilarity was detected between the experimental and control groups (the median times were 60 and 59 days, respectively, with *p* = 0.515).

In recent years, the watch‐and‐wait approach has come to the fore as a promising therapeutic alternative and has already been implemented in clinical practice. An increasing body of evidence regarding long‐term outcomes in patients managed without surgery is now available from various studies [11, 27‐29]. In our study, a greater number of patients in the experimental group attained cCR status than in the control group (16 and 5 patients in the experimental and control groups, respectively, with 5.9% vs. 1.9%, *p* = 0.024). In our research, more patients in the experimental group achieved cCR status than in the control group (16 vs. 5 patients, 5.9% vs. 1.9%, *p* = 0.024). Previous studies have indicated that the 2‐year local regrowth rate for patients with cCR under watch‐and‐wait ranges from 4.8% to 21% [26, 30]. Among those who experienced local regrowth, 95.4% underwent salvage therapy, with sphincter preservation achieved in 49.8% of patients who underwent salvage surgery [26]. In our study, another eight patients underwent a watch‐and‐wait approach and developed local recurrence (three in the experimental group and five in the control group), resulting in a local recurrence rate of 27.5%. All eight went on to have salvage surgery. The 2‐year local recurrence rate was 20.6% (with two patients experiencing local recurrence beyond 2 years), consistent with other reports.

This single‐center RCT presents a significant limitation. Patients from various medical centers, particularly those with expertise in treating rectal cancers, might have a more favorable prognosis. Surgeons with different learning curves were excluded from this trial. The generalization of our results to other medical centers and even other racial and ethnic groups demands further investigation.

In conclusion, the initial outcomes of this randomized controlled trial imply that the combination of four cycles of CapOX and pelvic radiation might increase the probability of attaining a complete pathological or clinical response in patients while the adverse effects remain within tolerable bounds. Particularly, the experimental treatment protocol has shown a higher pCR rate compared to the conventional treatment method. These discoveries merit additional research to validate the efficacy and safety of the protocol in a larger population.

## 4 Materials and Methods

### 4.1 Study Design and Participants

This single‐center, randomized, prospective, open‐label, phase III clinical trial (ClinicalTrials.gov Identifier: NCT02031939) was conducted at Sun Yat‐sen University Cancer Center. The primary objective was to compare four cycles of CapOX plus pelvic radiotherapy with standard capecitabine‐based radiotherapy. Ethical approval was obtained from the Ethics Committee of Sun Yat‐sen University Cancer Center (approval number: 5010‐2013‐012), and the research adhered to the principles outlined in the Declaration of Helsinki. The trial followed the Consolidated Standards of Reporting Trials (CONSORT) Guidelines [31]. All participants provided written informed consent before enrollment.

Eligible participants were adults aged 18–75 years with histologically confirmed rectal adenocarcinoma located within 10 cm of the anal verge. Pelvic MRI was used to evaluate clinical T and N stages, and eligible tumors included T3–T4 disease (with or without nodal involvement) or any T stage with N+ disease. Distant metastases were assessed by CT scans. Additional eligibility criteria required an Eastern Cooperative Oncology Group (ECOG) performance status of 0 or 1, as well as adequate organ function—specifically, normal bone marrow function and acceptable liver (based on normal aspartate aminotransferase, alkaline phosphatase, and alanine aminotransferase) and kidney function.

Exclusion criteria encompassed a history of malignancy other than adequately treated basal cell carcinoma of the skin or cervical carcinoma in situ; prior chemotherapy or pelvic radiotherapy; pregnancy or breastfeeding; known dihydropyrimidine dehydrogenase (DPD) deficiency; severe illnesses such as unstable angina or myocardial infarction within the previous 12 months; serious psychiatric disorders; emergency surgery for colorectal cancer complications (including bleeding, obstruction, or perforation); documented allergy to 5‐FU or DPD enzyme deficiency; participation in another randomized controlled trial within the past 3 months; and bone marrow suppression.

### 4.2 Randomization and Masking

Eligible participants were randomly allocated to receive radiotherapy plus four concurrent cycles of CapOX or capecitabine alone (CapRT). Randomization was performed in a 1:1 ratio by utilizing a computer‐generated schedule with block randomization and variable block sizes. To maintain allocation concealment and prevent the disclosure of the block sizes, the assignments were placed in sequentially numbered, sealed, opaque envelopes, which were then protected by two independent individuals.

### 4.3 Treatment Procedure

In the experimental group, patients underwent pelvic radiotherapy (50 Gy in 25 fractions) utilizing a 6–10 MV photon beam via intensity‐modulated radiation therapy in conjunction with four cycles of CapOX. The initial cycle (OXA 130 mg/m^2^, capecitabine 2000 mg/m^2^ from Days 1 to 14) was administered before radiotherapy as induction chemotherapy. The second and third cycles (OXA 100 mg/m^2^, capecitabine 2000 mg/m^2^ from Days 1 to 14) were provided concurrently with radiotherapy, and the fourth cycle (OXA 130 mg/m^2^, capecitabine 2000 mg/m^2^ from Days 1 to 14) was given as consolidation chemotherapy. In contrast, the control group underwent the same radiotherapy but received only capecitabine (825 mg/m^2^ twice daily, 5 days per week). The particulars of the treatment volumes can be found in the Supporting Information.

TME was mandatory, but the specific surgical approach (AR or abdominoperineal resection) and the decision regarding a temporary colostomy were at the surgeon's discretion. Other surgical methods (such as Hartmann's procedure, intersphincteric resection, or transanal local excision) were also allowed. Postoperatively, two additional cycles of CapOX and two cycles of capecitabine were suggested for patients in the experimental group, while six cycles of CapOX were recommended for those in the control group, regardless of pathological findings.

### 4.4 Pathology Procedure

Pathologists were unaware of each patient's treatment plan and independently assessed the surgical specimens. The response to treatment was classified based on the NCCN guidelines (from Grade 0: CR—“no viable cancer cells detected” to Grade 3: poor response—“minimal or no tumor reduction”) [32]. All resected lymph nodes, perineural invasion [33], and tumor deposits [34] were examined in accordance with standard procedures. All specimens were reviewed by two pathologists to guarantee reliable outcomes.

### 4.5 Watch‐and‐Wait Strategy

Approximately 6 weeks subsequent to radiotherapy, the patients underwent restaging via digital rectal examination (DRE), chest and abdominal CT, colonoscopy, as well as pelvic MRI. Those achieving cCR were presented with the watch‐and‐wait approach. These patients underwent all cycles of adjuvant chemotherapy and were meticulously monitored with DRE, endoscopy, chest and abdominal CT scans, and pelvic MRI every 3 months. cCR was defined by the absence of a discernible tumor during the digital examination, no detectable residual tumor observed through pelvic MRI or endoscopy, and the sustained absence of any residual tumor for a minimum duration of 12 months following CRT.

### 4.6 Surgical Complication Measurements

The incidence of morbidity and mortality occurring within a 30‐day period was assessed and recorded. The severity of all postoperative complications was evaluated using the Clavien‒Dindo classification [35].

### 4.7 Data Management and Statistical Analysis

The principal objective of this investigation was to assess the 5‐year OS rate. The research sought to evaluate an enhancement in the 5‐year OS, hypothesizing an increase from 72.4% in the control group to 77.4% in the experimental group. Based on this anticipated difference, a total of 556 eligible participants were required, assuming a two‐sided *α* of 0.05, a *β* of 0.1, and an expected dropout rate below 10%. Secondary endpoints included the pCR rate, AEs, quality of life assessments, tumor regression grading, sphincter preservation, surgical complications, and local control metrics.

Preoperative acute toxicities and surgical complications were documented following the National Cancer Institute Common Toxicity Criteria (version 4.0). Survival was measured from the point of randomization until an event or the most recent follow‐up. Local failure was classified as an event pertaining to local control, whereas tumor recurrence or death from cancer is considered an event related to DFS. Death from any cause was regarded as an event for OS. The pCR was identified as the complete lack of tumor cells at both the original tumor location and in the associated lymph nodes (ypT0N0). The cCR was indicated by the total absence of detectable tumors during DRE, colonoscopy, or imaging modalities, coupled with a persistent absence of any residual disease for a minimum of 12 months following CRT. Statistical analyses were conducted using chi‐square tests, Fisher's exact tests, and log‐rank tests as deemed appropriate, with a significance threshold established at *p* < 0.05.

## Author Contributions

X.J, Z.Z, Y.G, X.W., G.C., L.L., P.D., S.L., J.Z., M.L., Q.W., W.X., Z.P., and D.W. contributed to supervision, conceptualization, and project administration. Z.L., R.Z., F.W., H.W., and W.Z. composed and revised the manuscript. J.Z. and M.X.J. were responsible for data curation. H.W. and W. Z. contributed to data curation and data analysis. All authors have read and approved the final manuscript.

## Ethics Statement

This research conformed to the principles stipulated in the Declaration of Helsinki and received approval from the Ethics Committee of Sun Yat‐sen University Cancer Center (Approval No.: 5010‐2013‐012).

## Conflicts of Interest

The authors declare no conflicts of interest.

## Supporting information



Supporting Information

## Data Availability

The data supporting the conclusions of this research can be obtained from the corresponding author upon reasonable request.

## References

[1] W. Chen , R. Zheng , P. D. Baade , et al., “Cancer Statistics in China, 2015,” CA: A Cancer Journal for Clinicians 66, no. 2 (2016): 115–132. 26808342 10.3322/caac.21338

[2] C. Rodel , T. Liersch , R. Fietkau , et al., Preoperative Chemoradiotherapy and Postoperative Chemotherapy With 5‐Fluorouracil and Oxaliplatin Versus 5‐Fluorouracil Alone in Locally Advanced Rectal Cancer: Results of the German CAO/ARO/AIO‐04 Randomized Phase III Trial. (American Society of Clinical Oncology, 2014).

[3] J.‐P. Gérard , D. Azria , S. Gourgou‐Bourgade , et al., “Comparison of Two Neoadjuvant Chemoradiotherapy Regimens for Locally Advanced Rectal Cancer: Results of the Phase III Trial ACCORD 12/0405‐Prodige 2,” Journal of Clinical Oncology 28, no. 10 (2010): 1638–1644. 20194850 10.1200/JCO.2009.25.8376

[4] M. Roh , G. Yothers , M. O'Connell , et al., “The Impact of Capecitabine and Oxaliplatin in the Preoperative Multimodality Treatment in Patients With Carcinoma of the Rectum: NSABP R‐04,” Journal of Clinical Oncology 29, no. supplement S15 (2011): 3503–3503.21844503

[5] F. De Felice , A. L. Magnante , D. Musio , et al., “Diffusion‐Weighted Magnetic Resonance Imaging in Locally Advanced Rectal Cancer Treated With Neoadjuvant Chemoradiotherapy,” European Journal of Surgical Oncology 43, no. 7 (2017): 1324–1329. 28363512 10.1016/j.ejso.2017.03.010

[6] A. Body , H. Prenen , M. Lam , et al., “Neoadjuvant Therapy for Locally Advanced Rectal Cancer: Recent Advances and Ongoing Challenges,” Clinical Colorectal Cancer 20, no. 1 (2021): 29–41. 33531256 10.1016/j.clcc.2020.12.005

[7] Y. Deng , P. Chi , P. Lan , et al., “Modified FOLFOX6 With or Without Radiation Versus Fluorouracil and Leucovorin With Radiation in Neoadjuvant Treatment of Locally Advanced Rectal Cancer: Initial Results of the Chinese FOWARC Multicenter, Open‐Label, Randomized Three‐Arm Phase III Trial,” Journal of Clinical Oncology 34, no. 27 (2016): 3300–3307. 27480145 10.1200/JCO.2016.66.6198

[8] C. Aschele , L. Cionini , S. Lonardi , et al., “Primary Tumor Response to Preoperative Chemoradiation With or Without Oxaliplatin in Locally Advanced Rectal Cancer: Pathologic Results of the STAR‐01 Randomized Phase III Trial,” Journal of Clinical Oncology 29, no. 20 (2011): 2773–2780. 21606427 10.1200/JCO.2010.34.4911

[9] J.‐P. Gérard , D. Azria , S. Gourgou‐Bourgade , et al., “Clinical Outcome of the ACCORD 12/0405 PRODIGE 2 Randomized Trial in Rectal Cancer,” Journal of Clinical Oncology 30, no. 36 (2012): 4558–4565. 23109696 10.1200/JCO.2012.42.8771

[10] C. Rödel , T. Liersch , H. Becker , et al., “Preoperative Chemoradiotherapy and Postoperative Chemotherapy With Fluorouracil and Oxaliplatin Versus Fluorouracil Alone in Locally Advanced Rectal Cancer: Initial Results of the German CAO/ARO/AIO‐04 Randomised Phase 3 Trial,” Lancet Oncology 13, no. 7 (2012): 679–687. 22627104 10.1016/S1470-2045(12)70187-0

[11] J. Zhu , A. Liu , X. Sun , et al., “Multicenter, Randomized, Phase III Trial of Neoadjuvant Chemoradiation with Capecitabine and Irinotecan Guided by UGT1A1 Status in Patients With Locally Advanced Rectal Cancer,” Journal of Clinical Oncology 38, no. 36 (2020): 4231–4239. 33119477 10.1200/JCO.20.01932 PMC7768334

[12] R. R. Bahadoer , E. A. Dijkstra , B. van Etten , et al., “Short‐Course Radiotherapy Followed by Chemotherapy Before Total Mesorectal Excision (TME) Versus Preoperative Chemoradiotherapy, TME, and Optional Adjuvant Chemotherapy in Locally Advanced Rectal Cancer (RAPIDO): A Randomised, Open‐Label, Phase 3 Trial,” Lancet Oncology 22, no. 1 (2021): 29–42. 33301740 10.1016/S1470-2045(20)30555-6

[13] G. Chen , Y. Jin , W. L. Guan , et al., “Neoadjuvant PD‐1 Blockade With Sintilimab in Mismatch‐Repair Deficient, Locally Advanced Rectal Cancer: An Open‐Label, Single‐Centre Phase 2 Study,” Lancet Gastroenterology & Hepatology 8, no. 5 (2023): 422–431. 36870360 10.1016/S2468-1253(22)00439-3

[14] M. Mi , S. Weng , Z. Xu , H. Hu , Y. Wang , and Y. Yuan , “CSCO Guidelines for Colorectal Cancer Version 2023: Updates and Insights,” Chinese Journal of Cancer Research = Chung‐kuo Yen Cheng Yen Chiu 35, no. 3 (2023): 233–238. 37440826 10.21147/j.issn.1000-9604.2023.03.02 PMC10334498

[15] A. B. Benson , A. P. Venook , M. Adam , et al., “NCCN Guidelines® Insights: Rectal Cancer, Version 3.2024,” Journal of the National Comprehensive Cancer Network: JNCCN 22, no. 6 (2024): 366–375. 39151454 10.6004/jnccn.2024.0041

[16] H. Bando , Y. Tsukada , K. Inamori , et al., “Preoperative Chemoradiotherapy plus Nivolumab Before Surgery in Patients With Microsatellite Stable and Microsatellite Instability‐High Locally Advanced Rectal Cancer,” Clinical Cancer Research 28, no. 6 (2022): 1136–1146. 35063964 10.1158/1078-0432.CCR-21-3213 PMC9365382

[17] A. Shamseddine , Y. H. Zeidan , Z. El Husseini , et al., “Efficacy and Safety‐In Analysis of Short‐Course Radiation Followed by mFOLFOX‐6 Plus Avelumab for Locally Advanced Rectal Adenocarcinoma,” Radiation Oncology 15, no. 1 (2020): 233. 33028346 10.1186/s13014-020-01673-6 PMC7542723

[18] W. W. Xiao , G. Chen , Y. H. Gao , et al., “Effect of Neoadjuvant Chemoradiotherapy With or Without PD‐1 Antibody Sintilimab in pMMR Locally Advanced Rectal Cancer: A Randomized Clinical Trial,” Cancer Cell 42, no. 9 (2024): 1570–1581.e4. 39094560 10.1016/j.ccell.2024.07.004

[19] R. Glynne‐Jones , L. Wyrwicz , E. Tiret , et al., “Rectal Cancer: ESMO Clinical Practice Guidelines for Diagnosis, Treatment and Follow‐Up,” Annals of Oncology 28 (2017): iv22–iv40. 28881920 10.1093/annonc/mdx224

[20] J. Garcia‐Aguilar , S. Patil , J. K. Kim , et al., Preliminary Results of the Organ Preservation of Rectal Adenocarcinoma (OPRA) Trial. (American Society of Clinical Oncology, 2020).

[21] O. S. Guner and L. V. Tumay , “Persistent Extramural Vascular Invasion Positivity on Magnetic Resonance Imaging After Neoadjuvant Chemoradiotherapy Predicts Poor Outcome in Rectal Cancer,” Asian Journal of Surgery 44, no. 6 (2021): 841–847. 33573925 10.1016/j.asjsur.2021.01.011

[22] W. J. Mei , X. Z. Wang , Y. F. Li , et al., “Neoadjuvant Chemotherapy With CAPOX Versus Chemoradiation for Locally Advanced Rectal Cancer With Uninvolved Mesorectal Fascia (CONVERT): Initial Results of a Phase III Trial,” Annals of Surgery 277, no. 4 (2023): 557–564. 36538627 10.1097/SLA.0000000000005780 PMC9994847

[23] D. Schrag , Q. Shi , M. R. Weiser , et al., “Preoperative Treatment of Locally Advanced Rectal Cancer,” New England Journal of Medicine 389, no. 4 (2023): 322–334. 37272534 10.1056/NEJMoa2303269 PMC10775881

[24] J. H. Lefevre , L. Mineur , S. Kotti , et al., “Effect of Interval (7 or 11 Weeks) Between Neoadjuvant Radiochemotherapy and Surgery on Complete Pathologic Response in Rectal Cancer: A Multicenter, Randomized, Controlled Trial (GRECCAR‐6),” Journal of Clinical Oncology 34, no. 31 (2016): 3773–3780. 27432930 10.1200/JCO.2016.67.6049

[25] E. Akgun , C. Caliskan , O. Bozbiyik , et al., “Randomized Clinical Trial of Short or Long Interval Between Neoadjuvant Chemoradiotherapy and Surgery for Rectal Cancer,” Journal of British Surgery 105, no. 11 (2018): 1417–1425. 10.1002/bjs.10984 30155949

[26] É. J. Ryan , D. O'Sullivan , M. Kelly , et al., “Meta‐Analysis of the Effect of Extending the Interval After Long‐Course Chemoradiotherapy Before Surgery in Locally Advanced Rectal Cancer,” Journal of British Surgery 106, no. 10 (2019): 1298–1310. 10.1002/bjs.11220 31216064

[27] A. Habr‐Gama , R. O. Perez , W. Nadalin , et al., “Operative Versus Nonoperative Treatment for Stage 0 Distal Rectal Cancer Following Chemoradiation Therapy: Long‐Term Results,” Annals of Surgery 240, no. 4 (2004): 711. 15383798 10.1097/01.sla.0000141194.27992.32 PMC1356472

[28] R. G. H. Beets‐Tan , J. Leijtens , and G. L. Beets , “Wait‐and‐See Policy for Clinical Complete Responders After Chemoradiation for Rectal Cancer,” Journal of Clinical Oncology 29, no. 35 (2011): 4633–4640. 22067400 10.1200/JCO.2011.37.7176

[29] A. L. Appelt , J. Pløen , H. Harling , et al., “High‐Dose Chemoradiotherapy and Watchful Waiting for Distal Rectal Cancer: A Prospective Observational Study,” Lancet Oncology 16, no. 8 (2015): 919–927. 26156652 10.1016/S1470-2045(15)00120-5

[30] J. J. Smith , P. Strombom , O. S. Chow , et al., “Assessment of a Watch‐and‐Wait Strategy for Rectal Cancer in Patients With a Complete Response After Neoadjuvant Therapy,” JAMA Oncology 5, no. 4 (2019): e185896–e185896. 30629084 10.1001/jamaoncol.2018.5896 PMC6459120

[31] K. F. Schulz , D. G. Altman , and D. Moher , “CONSORT 2010 Statement: Updated Guidelines for Reporting Parallel Group Randomised Trials,” BMJ 340 (2010): c332. 20332509 10.1136/bmj.c332 PMC2844940

[32] M. Gavioli , G. Luppi , L. Losi , et al., “Incidence and Clinical Impact of Sterilized Disease and Minimal Residual Disease After Preoperative Radiochemotherapy for Rectal Cancer,” Diseases of the Colon & Rectum 48, no. 10 (2005): 1851–1857. 16132481 10.1007/s10350-005-0133-6

[33] S. Fujita , T. Shimoda , K. Yoshimura , S. Yamamoto , T. Akasu , and Y. Moriya , “Prospective Evaluation of Prognostic Factors in Patients With Colorectal Cancer Undergoing Curative Resection,” Journal of Surgical Oncology 84, no. 3 (2003): 127–131. 14598355 10.1002/jso.10308

[34] I. Nagtegaal and P. Quirke , “Colorectal Tumour Deposits in the Mesorectum and Pericolon; a Critical Review,” Histopathology 51, no. 2 (2007): 141–149. 17532768 10.1111/j.1365-2559.2007.02720.x

[35] D. Dindo , N. Demartines , and P.‐A. Clavien , “Classification of Surgical Complications: A New Proposal With Evaluation in a Cohort of 6336 Patients and Results of a Survey,” Annals of Surgery 240, no. 2 (2004): 205. 15273542 10.1097/01.sla.0000133083.54934.ae PMC1360123

