# Supplementary material for: Short‐Term Results of the SONCAR Study: Optimized Neoadjuvant Chemoradiotherapy in Locally Advanced Rectal Cancer Patients
Source: MedComm (2020). 2025 Jul 23;6(8):e70222. doi: 10.1002/mco2.70222 (PMC12284437; doi:10.1002/mco2.70222)
Supplement: Supplementary file 1 — Supporting Information [file MCO2-6-e70222-s001.docx]

| **Table S1. Treatment compliance among patients who received preoperative chemoradiotherapy** | | | |
| --- | --- | --- | --- |
| **Treatment Compliance** | **Treatment Group, No. (%)** | | ***P* value** |
|  | **Experimental Group**  **(n = 269)** | **Control Group**  **(n = 267)** |  |
| Induction chemotherapy |  |  | <0.001 |
| CapOX | 258 (95.9) | 1 (0.4) |  |
| Capecitabine | 1 (0.4) | 4 (1.5) |  |
| None | 10 (3.7) | 262 (98.1) |  |
| Radiation |  |  | 0.890 |
| Full dose (≥ 50Gy) | 257 (95.5) | 252 (94.4) |  |
| ≥ 90% of planned | 9 (3.3) | 11 (4.1) |  |
| < 90% of planned | 2 (0.7) | 2 (0.7) |  |
| None | 1 (0.4) | 2 (0.7) |  |
| Chemotherapy during radiotherapy |  |  | <0.001 |
| CapOX | 255 (94.8) | 5 (1.9) |  |
| Capecitabine | 12 (4.5) | 260 (97.4) |  |
| None | 2 (0.7) | 2 (0.7) |  |
| Cycles of chemotherapy during radiotherapy |  |  | 0.722 |
| 0 | 2 (0.7) | 2 (0.7) |  |
| 1 | 22 (8.2) | 17 (6.4) |  |
| 2 | 245 (91.1) | 248 (92.9) |  |
| Consolidation chemotherapy |  |  | <0.001 |
| CapOX | 230 (85.5) | 23 (8.6) |  |
| Capecitabine | 0 | 3 (1.1) |  |
| None | 39 (14.5) | 241 (90.3) |  |
| Adjuvant chemotherapy |  |  | <0.001 |
| CapOX | 69 (25.7) | 142 (53.2) |  |
| Capecitabine | 25 (9.3) | 17 (6.4) |  |
| Other (CapOX+Capecitabine) | 94 (34.9) | 21 (7.9) |  |
| None | 81 (30.1) | 87 (32.6) |  |
| Cycles of adjuvant Chemotherapy |  |  | <0.001 |
| 0 | 81 (30.1) | 87 (32.6) |  |
| 1 | 16 (5.9) | 12 (4.5) |  |
| 2 | 44 (16.4) | 5 (1.9) |  |
| 3 | 21 (7.8) | 12 (4.5) |  |
| 4 | 97 (36.1) | 21 (7.9) |  |
| 5 | 8 (3.0) | 20 (7.5) |  |
| 6 | 2 (0.7) | 110 (41.2) |  |
| Time gap between nCRT and surgery (days) |  |  | 0.515 |
| Mean (SD) | 65.1 (1.6) | 67.2 (3.3) |  |
| Median (Range) | 60 (40-222) | 59 (35-604) |  |
| Abbreviation: nCRT, neoadjuvant chemoradiotherapy; SD, standard deviation. | | | |

| **Table S2. Patient characteristics after chemoradiotherapy** | | | |
| --- | --- | --- | --- |
| **Characteristic** | **Treatment Group, No. (%)** | | ***P* value** |
|  | **Experimental Group**  **(n = 269)** | **Control Group**  **(n = 267)** |  |
| Clinical T category |  |  | 0.050 |
| cT0 | 5 (1.9) | 0 |  |
| cT1 | 1 (0.4) | 6 (2.2) |  |
| cT2 | 19 (7.1) | 23 (8.6) |  |
| cT3 | 137 (50.9) | 130 (48.7) |  |
| cT4 | 56 (20.8) | 48 (18.0) |  |
| Missing | 51 (19.0) | 60 (22.5) |  |
| Clinical N category |  |  | 0.668 |
| cN0 | 64 (23.8) | 69 (25.8) |  |
| cN1 | 114 (42.4) | 101 (37.8) |  |
| cN2 | 40 (14.9) | 37 (13.9) |  |
| Missing | 51 (19.0) | 60 (22.5) |  |
| Distance from the anal verge, cm |  |  | 0.309 |
| ≤ 5 cm | 136 (50.6) | 127 (47.6) |  |
| > 5 cm | 93 (34.6) | 107 (40.1) |  |
| Missing | 40 (14.9) | 33 (12.4) |  |
| MRF |  |  | 0.840 |
| Positive | 67 (24.9) | 64 (24.0) |  |
| Negative | 193 (71.7) | 196 (73.4) |  |
| Not reported | 7 (2.6) | 4 (1.5) |  |
| Levator ani involved |  |  | .997 |
| Yes | 0 | 1 (0.4) |  |
| No | 269 (100.0) | 266 (99.6) |  |
| External sphincter muscle of anus involved |  |  | >0.999 |
| Yes | 2 (0.7) | 2 (0.7) |  |
| No | 267 (99.3) | 265 (99.3) |  |
| EMVI |  |  | 0.812 |
| Positive | 59 (21.9) | 63 (23.6) |  |
| Negative | 201 (74.7) | 200 (74.9) |  |
| Unknown or missing | 9 (3.3) | 4 (1.5) |  |
| Regional LN |  |  | 0.434 |
| Positive | 241 (86.4) | 230 (89.8) |  |
| Negative | 36 (12.9) | 24 (9.4) |  |
| Unknown or missing | 2 (0.7) | 2 (0.8) |  |
| Abbreviation: MRF, mesorectal fascia; EMVI, extramural vascular invasion; LN, lymph nodes. | | | |

| **Table S3. Subgroup analysis of rectal cancer patients according to the distance from the lower edge of the tumor to the anal verge** | | | | | | |
| --- | --- | --- | --- | --- | --- | --- |
| **Variable** | **Below 5 cm from anal verge** | | ***P* value** | **Above 5 cm from anal verge** | | ***P* value** |
|  | **Treatment Group, No. (%)** | |  | **Treatment Group, No. (%)** | |  |
|  | **Experiment group**  **(n = 145)** | **Control group**  **(n = 134)** |  | **Experiment group**  **(n = 87)** | **Control group**  **(n = 107)** |  |
| Tumor regression grade |  |  | 0.024 |  |  | 0.305 |
| TRG0 | 49 (33.8) | 29 (21.6) |  | 26 (29.9) | 25 (23.4) |  |
| None TRG0 | 96 (66.2) | 105 (78.4) |  | 61 (70.1) | 82 (76.6) |  |
| Pathological complete response |  |  | 0.024 |  |  | 0.181 |
| pCR | 49 (33.8) | 29 (21.6) |  | 26 (29.9) | 23 (21.5) |  |
| Non pCR | 96 (66.2) | 105 (78.4) |  | 61 (70.1) | 84 (78.5) |  |
| Pathological T category |  |  | 0.024 |  |  | 0.305 |
| ypT0 | 49 (33.8) | 29 (21.6) |  | 26 (29.9) | 25 (23.4) |  |
| None ypT0 | 96 (66.2) | 105 (78.4) |  | 61 (70.1) | 82 (76.6) |  |
| Pathological N category |  |  | 0.514 |  |  | 0.124 |
| ypN0 | 118 (81.4) | 113 (84.4) |  | 77 (88.5) | 86 (80.4) |  |
| None ypN0 | 27 (18.6) | 21 (15.7) |  | 10 (11.5) | 21 (19.6) |  |
| ypT0-1N0M0 |  |  | 0.248 |  |  | 0.818 |
| Yes | 55 (37.9) | 42 (31.3) |  | 29 (33.3) | 34 (31.8) |  |
| No | 90 (62.1) | 92 (68.7) |  | 58 (66.7) | 73 (68.2) |  |
| Abbreviation: TRG, tumor regression grade; pCR, pathological complete response. | | | | | | |
